# Multiple isolated extramedullary relapse of acute promyelocytic leukemia after allogeneic hematopoietic stem cell transplant: a case report and review of literature

**DOI:** 10.1186/2193-1801-2-49

**Published:** 2013-02-12

**Authors:** Shalin Kothari, Geoffrey Herzig, Stephen Slone, Roger Herzig

**Affiliations:** Division of Blood and Bone Marrow Transplant, University of Louisville, James Graham Brown Cancer Center, 529 South Jackson Street, Louisville, KY 40202 USA; Department of Pathology and Laboratory Medicine, University of Louisville, Louisville, KY 40292 USA

## Abstract

Isolated extramedullary disease (EMD) is uncommon, especially in acute promyelocytic leukemia (APL) after allogeneic hematopoietic stem cell transplantation (HSCT). We review the literature and present a 32 year old woman with APL who developed multiple EMDs after allogeneic HSCT within the calvarium, and later found to have various isolated lesions including femur, humerus and thoraco lumbar vertebrae. She was treated with local radiotherapy (XRT) to EMD lesions, all-trans retinoic acid, arsenic trioxide and donor lymphocyte infusion at different time points in her clinical course, without success. Out of reported cases in clinical setting as ours, average onset of isolated EMD is 25 months and median survival 14 months. Effective treatment of isolated EMD after HSCT is not yet clear, but ATO in combination with local XRT, tamibarotene and second HSCT have shown good results in some reported cases, but accumulation of more cases is needed to elucidate optimal therapy in such setting.

**Electronic supplementary material:**

The online version of this article (doi:10.1186/2193-1801-2-49) contains supplementary material, which is available to authorized users.

## Introduction

Among the more treatable subtypes of acute myeloid leukemia (AML) is acute promyelocytic leukemia (APL). Nearly all patients with APL have a translocation of chromosomes 15 and 17, resulting in the fusion between RARα (retinoic acid receptor α), which encodes a retinoic acid receptor, and the promyelocytic leukemia (PML) protein (Licht 2009). “Myeloid sarcoma”, a solid extramedullary mass composed of immature myeloid cells, was first described by Burns in 1811 (Burns 1823). King (1853) used the term “chloroma” because of its greenish appearance, caused by myeloperoxidase in the leukemic cells (King 1853). Although myeloid sarcomas usually appear as a manifestation of systemic leukemias, they occasionally precedea marrow relapse and may be the presenting finding (Mwanda and Rajab 1999). We present a case of multiple skull lesions of myeloid sarcoma in a patient with APL that occurred seven months after allogeneic hematopoietic stem cell transplant (HSCT). For the rest of this review, myeloid sarcomas will be referred to as extramedullary disease (EMD).

## Case report

The patient, a 32 year-old woman, was diagnosed with APL (bcr1 subtype) and treated at an area hospital with standard oral all-trans retinoic acid (ATRA), cytarabine and idarubicin induction with initial complete remission (CR). She maintained CR status for 18 months with 2 courses of consolidation therapy consisting of idarubicin and cytarabine. However, she relapsed in BM and CNS while being on ATRA as maintenance therapy. She had a successful re-induction with arsenic trioxide (ATO) and intrathecal cytarabine followed by high-dose busulfan and etoposide with autologous PML/RARα PCR-negative hematopoietic cells rescue. She relapsed in 7 months and was referred to our institution for an allogeneic HSCT from her HLA matched sister after cyclophosphamide and total body irradiation (TBI) conditioning regimen. She achieved full donor chimerism with no molecular evidence of disease. Seven months later, she presented with right fronto-parietal headaches and also noticed a “bump” over the right frontal area.

**Figure 1:**
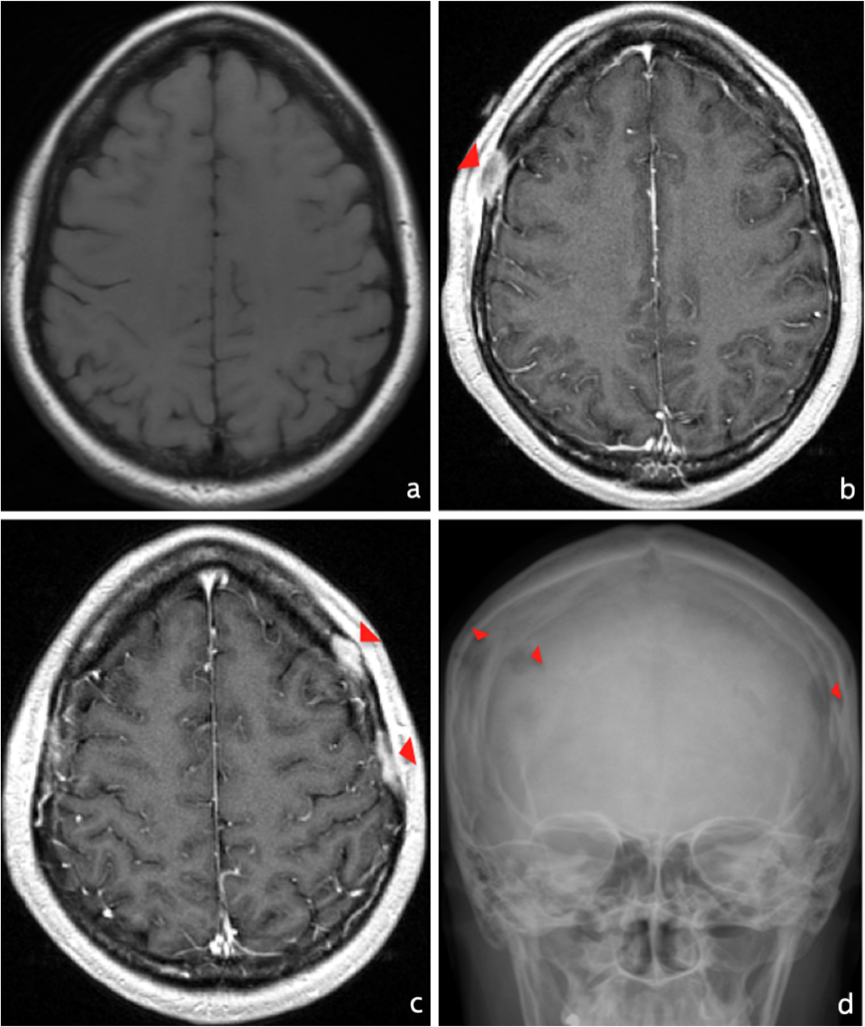
**a- Normal pre-allogeneic HSCT MRI of brain.b** and **c**- Post contrast 3D spoiled gradient echo showing numerous thickened contrast enhancing lesions measuring up to 1.6 × 0.8 cm in the left frontal, 2.0 × 0.9 cm in the right frontal and 2.7 × 0.5 cm in the left parietal region, within the calvarium. **d**- X-ray of skull, frontal view showing lytic lesions in the frontal and parietal bones.

Magnetic resonance imaging (MRI) showed multiple enhancing lesions within the calvarium, with most prominent lesion in right frontal bone, measuring 2.0 × 0.9 cm that were not present on MRI done before allogeneic HSCT (Figure 1). The lesions were iso- to slightly hypointense on T1 and isointense on T2-weighted images. Subsequent skeletal survey and whole body PET/CT imaging showed multiple bony lesions involving frontal and parietal bones, bilateral glenoid fossae, proximal left femur, distal left humerus and T5, T9 and L4 vertebral bodies.

Biopsy of right frontal lesion revealed EMD that expressed CD45 (dim to moderate), CD34 and CD117 (focal weak); and did not express CD56 and CD68. Fluorescent in-situ hybridization (FISH) identified 22 out of 50 cells positive for PML/RARα translocation. It was clear that these EMD lesions were secondary to her APL. Iliac crest bone marrow biopsy showed no morphologic evidence of leukemia and chimerism evaluation by microsatellite polymorphisms showed 100% of cells of donor origin; peripheral blood PCR for PML/RARA transcript was negative.

Post-transplant immunosuppression was discontinued; she was treated with ATRA, intrathecal methotrexate, and radiation therapy (XRT) to skull and proximal left femur. After radiation therapy (a total of 20 Gy in 10 fractions), she received two months of ATO, but had progressive disease evident on whole body PET/CT imaging. She was then treated with high-dose cytarabine and idarubicin with infusion of stored donor hematopoietic cells (DLI), but died of complications 16 days after starting therapy. CT scan of head without contrast done day the before her death re-demonstrated the skull lesions.

**Figure 2:**
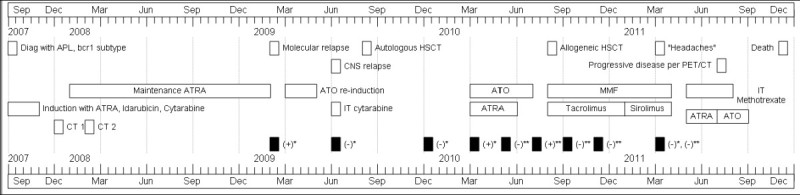
**Timeline of course of disease and treatment.** - PML/RARα transcript. *- in bone marrow by PCR, **- in peripheral blood. Diag- Diagnosed, APL- Acute promyelocytic leukemia, HSCT- Hematopoietic stem cell transplantation, dz- disease, CNS- Central nervous system, ATRA- All-trans retinoic acid, ATO- Arsenic trioxide, MMF- Mycophenolate mofetil, IT- Intra-thecal, CT- Consolidation therapy.

Figure 2 includes a summary of clinical course of our patient.

## Discussion

In this report, we present a patient with CD56 negative APL after allogeneic HSCT who developed multiple EMD without BM relapse (isolated EMD) early in her post-transplant course. She was treated with local XRT to EMD lesions, ATRA, ATO and DLI at different points of time, without success.

In general, isolated EMD of acute myeloid leukemia (AML), following allogeneic HSCT is uncommon, and occurs in about 0.65% of all HSCT cases as estimated from the European BM Transplant Registry, with higher rates of up to 10% reported in long-term survivors (Bekassy et al. 1996). In contrast to our patient, who remained in molecular and hematologic remission for five months after isolated EMD, most isolated extramedullary relapses portend BM relapse (Ruiz-Arguelles et al. 2005).

There have been 12 reported cases, including ours, of isolated EMD with APL after HSCT (Table 1). Among them, reported sites of EMD are skin, testes, bone, CNS, spine, psoas muscle, middle ear, scalp, breast, nasal cavity, pharynx and bowel. There is no obvious conclusion to site predilection.

**Table 1:** **Reported cases of acute promyelocytic leukemia with EMD after HSCT**

Case report	TBI-containing conditioning regimen?	Onset of EMD after HSCT	Treatment tried at various time points for EMD	BM status after treatment	Patient status after EMD
(Bekassy et al. 1996)	No	54 mo	Local XRT, surgical resection, CT*	Relapse after 5 mo	Alive at 15 mo
	Yes	28 mo	Local XRT, surgical resection, CT*	Relapse after 11 mo	Death at 14 mo
	No	20 mo	Local XRT, CT*	Relapse after 1 mo	Death at 1 mo
(Forrest et al. 1997)	No	48 mo	Local XRT, ATRA, Ara-C, DNR	Remission	Death at 25 mo
(Milone et al. 1999)	No	9 mo	Etoposide, whole skin XRT, DLI	Remission	NM
(Ustun et al. 2001)	NM	13 mo	ATRA, local XRT	Remission	Alive at 19 mo
(Classen et al. 2003)	No	18 mo	DNR; ATRA; Topotecan; intrathecal MTX, prednisolone, Ara-C; 2^nd^ HSCT	Remission	Alive at 43 mo
(Ammatuna et al. 2005)	NM	36 mo	GO, Local XRT, DLI, ATO	Relapse after 7 mo	Alive at 13 mo
(Kai et al. 2006)	Yes	14 mo	ATO, Local XRT	Remission	Alive at 20 mo
(Naina et al. 2011)	No	31 mo	GO, ATRA, tamibarotene	Remission	Alive at 12 mo
(Ochs et al. 2010)	No	25 mo	Surgical resection, ATRA, Local XRT	Relapse “shortly”	Death at 4 mo
Our case	Yes	7 mo	ATRA, intrathecal MTX, local XRT, ATO, DLI	Remission	Death at 8 mo

*TBI*- Total-body irradiation, *EMD*- Extramedullary disease, *HSCT*- Hematopoietic stem cell transplantation, *BM*- Bone marrow, *XRT*- radiotherapy, CT*- Specifics of chemotherapy not described, mo- months, *ATRA*- All-trans retinoic acid, *Ara-C*- Cytarabine, *DNR*- Daunorubicin, *DLI*- Donor lymphocyte infusion, *NM*- Not mentioned, *MTX*- Methotrexate, *GO*- Gemtuzumab ozogamicin, *ATO*- Arsenic trioxide.

Average onset of EMD after HSCT in APL patients is 19 months, ranging from 7 months to 54 months. Our patient presented within 7 months of allogeneic HSCT- the earliest of reported cases with EMD. Significance of such wide range of onset remains unknown.

There are no known risk factors for EMD (Kai et al. 2006), but several have been postulated. Some authors have suggested an increase in the incidence of EMD since the introduction of ATRA in the treatment of APL. Modulation of APL blasts and endothelial cells adhesion molecules, and upregulation of the granulocyte-colony stimulating factor receptor are some of the proposed mechanisms (de Botton et al. 2006; Weiss and Warrell 1994; Wiernik et al. 1996). The expression of CD56 has been associated with an increase in EMD and resistance to ATRA and anthracycline therapy (Montesinos et al. 2011). The role of ATRA in the development of EMD in APL remains controversial, especially in CD56 negative EMD, as in our case (Ito et al. 2004). Given the universal use of ATRA in APL and the rarity of EMDs, ATRA seems to have only a small role, if any, in development of EMDs. Kai et al. speculated that non-TBI containing regimens might increase the risk for EMD after HSCT in APL (Kai et al. 2006). Since only three of the reported cases received TBI-containing regimen, more cases need to be analyzed to elucidate the role of conditioning regimen as a risk factor for EMD. It has also been suggested that certain ethnic groups may be at higher risk of EMD in APL (Wiernik et al. 1996). Some investigators suggested that tissue injury caused by diagnostic procedures and bleeding leads to occurrence of EMD via leakage of leukemic cells, growth factors, and cell mediators (Sanz et al. 2000). Except for ATRA, our patient did not have any of these proposed risk factors.

Biopsy is the most preferred diagnostic method for EMD. Our biopsy result differs from the observation of Pileri et al. and Campidelli et al. (Campidelli et al. 2009; Pileri et al. 2007), in that, EMD, in our case, did not express CD68/KP1. The vast majority of normal CD34 positive hematopoietic precursors are CD68 negative, whereas AML CD34 positive blasts are usually CD68 positive. CD68 epitopes have been suggested as a potential target for leukemia-reactive cytotoxic-T cell (Sadovnikova et al. 2002). But, patients such as ours may not benefit from such therapy.

Bony EMD lesions appear lytic rather than sclerotic (Libson et al. 1986; Cho et al. 1990). On MRI, they are typically iso- to slightly hypointense on T1- and isointense on T2-weighted images relative to brain tissue (Kao et al. 1987). Both CT scan and MRI are diagnostically very useful, but MRI better reveals the exact extent of the tumor and the potential invasion of the cranial nerve or a intracranial structure (Chang et al. 2009).

As evident from Table 1, BM status after EMD has little influence on patient survival. Our patient remained in CR and showed 100% donor chimerism even though she succumbed to her progressive disease within 8 months.

During the course of APL, recognition of EMD is important as aggressive induction chemotherapy or XRT can induce CR. Several treatment options for isolated EMD after allogeneic HSCT in APL patients have been postulated, which include local XRT, ATRA, tamibarotene (earlier known as Am80), ATO, DLI, or a second allogeneic HSCT (Kai et al. 2006). Successful treatment was documented with gemtuzumab ozogamicin (Mylotarg) by many authors before FDA withdrew it in 2010 (Cohen et al. 2002; Ando et al. 2010). Treatment tried in the reported cases of post-HSCT EMDs in APL patients are summarized in Table 1. The combination therapy of ATO and XRT for APL EMDs may be reasonable, since enhancement of sensitivity to XRT by ATO treatment has been reported for some kind of solid tumors (Griffin et al. 2005), though, as evident from Table 1, treatment with ATO and local XRT has not been fully explored in such setting. The longest survivor had a second allogeneic HSCT, suggesting that this approach may be the only curative one.

Although long-term survival has occasionally been achieved, the prognosis for EMD patients is generally poor, particularly when they occur during remissions and after HSCT (Bekassy et al. 1996). Median survival after EMD in such setting is 14 months (range: 1–43 months).

## Conclusion

EMD is uncommon, especially in APL after treatment. Average onset of isolated EMD after HSCT in APL patients is 25 months. The role of ATRA in development of EMDs needs to be elucidated, especially in CD56 negative cases. Despite graft versus host disease and the potential graft versus leukemia effect, our patient had EMD and maintained 100% donor chimerism. In setting as our patient’s, BM status after EMD has little influence, if at all, in overall patient survival. There is a potential for research in novel therapies targeting surface markers, especially in CD68 negative myeloid cells. Treatment of isolated EMD after HSCT remains a challenge. ATO and local XRT have shown good response in some reports but a second allogeneic HSCT may be needed for long-term survival.

## Authors’ original submitted files for images

Below are the links to the authors’ original submitted files for images. Authors’ original file for figure 1 Authors’ original file for figure 2
