# Supplementary figures and images for: Multiple isolated extramedullary relapse of acute promyelocytic leukemia after allogeneic hematopoietic stem cell transplant: a case report and review of literature
Source: Springerplus. 2013 Feb 12;2(1):49. doi: 10.1186/2193-1801-2-49 (PMC3586393; doi:10.1186/2193-1801-2-49)

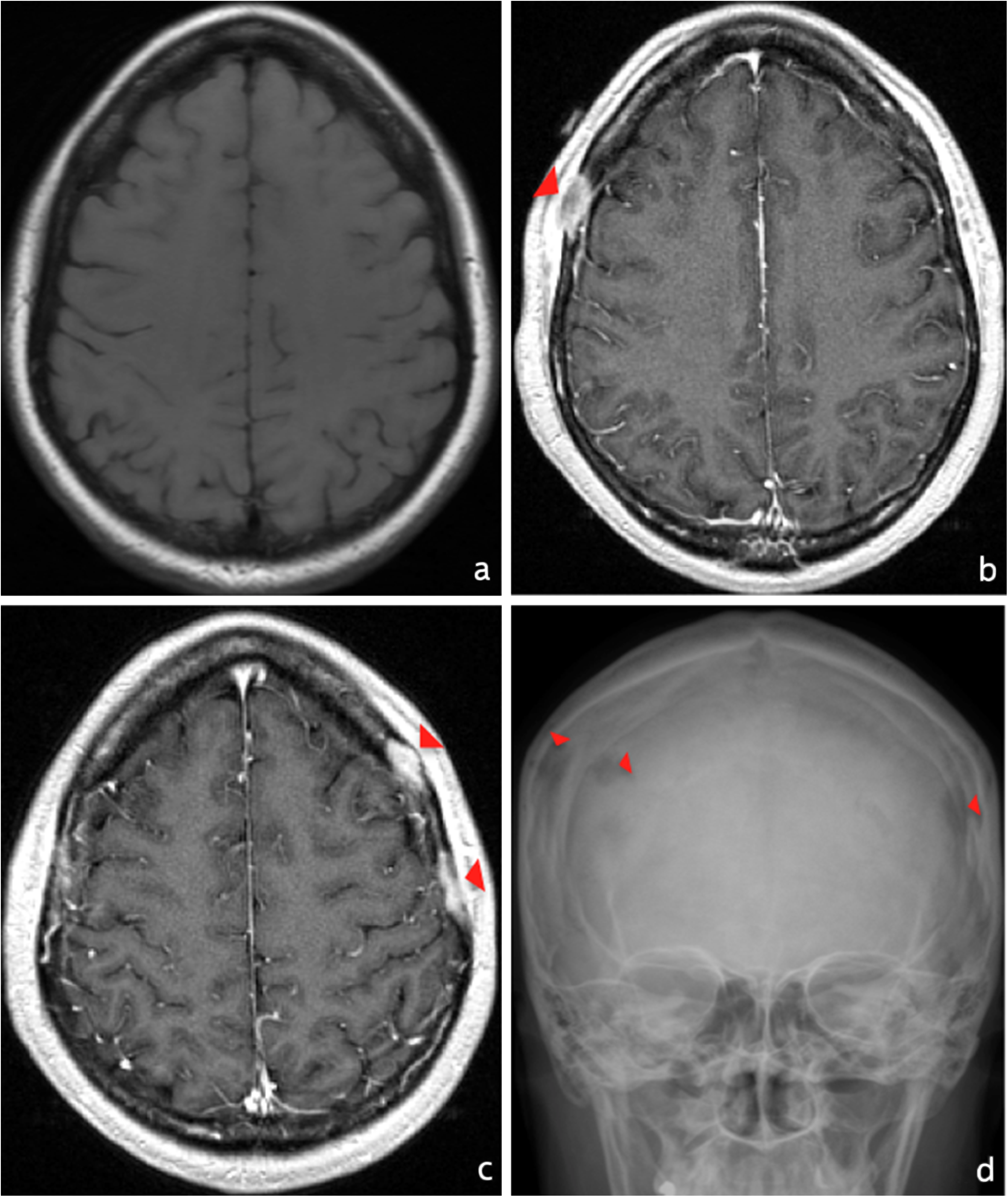

Supplement: Supplementary file 1 — Authors’ original file for figure 1 [file 40064_2012_100_MOESM1_ESM.tiff]

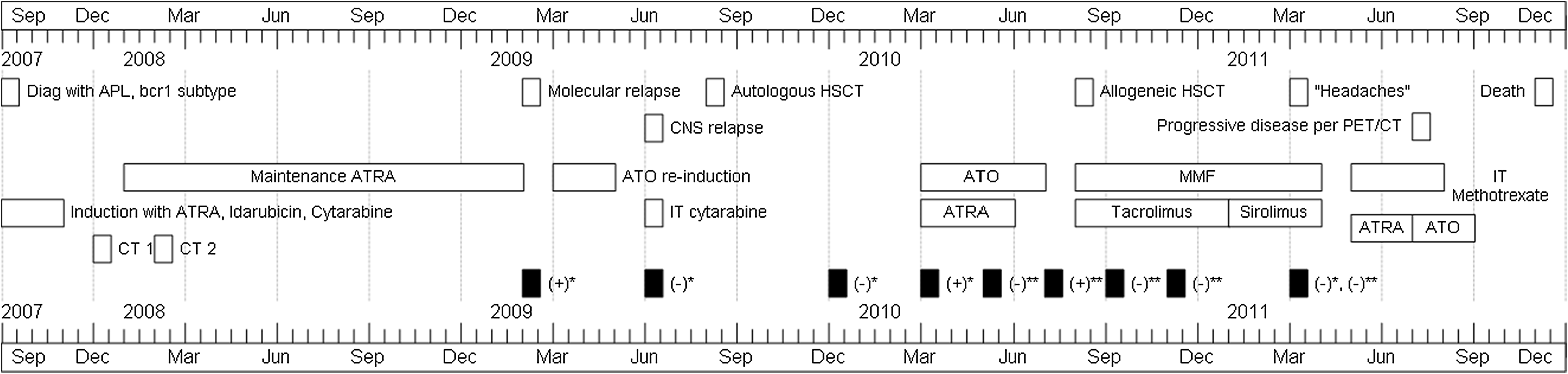

Supplement: Supplementary file 2 — Authors’ original file for figure 2 [file 40064_2012_100_MOESM2_ESM.tiff]
